# Culture

**Published:** 1884-01

**Authors:** J. B. Chandler


### CULTURE.

J. B. CHANDLER.

Culture, says Mathew Arnold in one of his books, entitled "Literature and Dogma," is eminently necessary for the proper understanding of any book, even the Bible, and culture he defines as "the acquainting ourselves with the best that has been known and said in the world."

In the culture of the earth we find the sturdy farmer apportioning out his new land, clearing off, testing the soil to find its capabilities and wants, then plowing and planting. The seed once in the earth the wonderful mystery of its growth lies with a Higher Power than the planter; yet he has learned from experience that under certain circumstances, with care to keep down intruding weeds, the young shoots will grow vigorously, and a good harvest will follow.

Human culture has a parallel. The careful parent selects a school which will fit his child to bear the bright fruit in the future he so ardently desires. All that can be acquired there is but preparing the soil and planting the seed. The mystery of acquiring, or understanding, of applying, lies in the will and brain of the student, under that same Higher Power which permits the growth of the seed planted by the husbandman.

The highest graduate from the best college in the world falls far short of Mr. Arnold's definition of culture. Given that he had the best teachers, whose efforts he has supplemented with careful study, he leaves the Alma Mater with only the seed planted. His training has fitted him to begin; he has within him that from which culture starts, the seed from which, with care, is to spring the ability to acquire and improve. Like the husbandman, his harvest is only to be reached through constant watching and careful culture. There are many vicious and noxious weeds to fear, which, having once secured a foothold, are difficult to uproot. But starting with the will fixed upon success in the pursuit of culture, under judicious advice the student soon becomes fitted to select the true path himself, when improvement and pleasure go hand in hand.

Few can become admirable Crichtons, excelling in everything, yet there are few who will not be able to reach the levels of culture from which they can look forth with pleasure and sound judgment, combined with acquired good taste. Knowledge is power, and its acquirement is necessarily such a training of mind and body that refinement, sound judgment and good taste must naturally be its accompaniments. I refer to general knowledge, not the mere attributes of a specialty; the acquainting ourselves as much as possible with "the best that has been known and said."

The habit of, and taste for good reading once formed, all the capabilities of mind and body for enjoyment may be reached. In the pursuit of culture the bright and beautiful avenues of belles letters are to be explored as well as the depths of history and science. Nor would I shut out the works of those who stand acknowledged as exponents of the best romance. The taste for art must be cultivated and word painting, with character sculpture must be studied equally with the works from the brush or the chisel. But ever let the definition and aim be kept in mind—the *best* that has been said and done. The short road to culture is in the avoidance, as much as possible, of all but the best. There are depths of shadows and brilliant high lights enough for contrast even in the best pictures. Excuses are made by many that the actual duties of life preclude the cultivation of the mind. Mark the career of any man who makes his business his life, who can find no time for aught save sleeping, eating and working A mind narrowed to the contemplation of accumulating pennies; a brain softened by the pressure of one idea; possibly an early death from the unnatural concentration of body and mind upon a single object. Or, should pecuniary success follow his sacrifices and labors, old age finds him surrounded with luxuries and means which have no charms because he has no tastes to enjoy the one or to gratify with the other; he sinks friendless into the grave regretting nothing but the loss of the counting room.

Contrast such age with that of one who has, through life, whilst providing for the body, catered to the wants of the mind. Business has had the proper share of the day; the pleasure of acquiring knowledge and developing tastes have had their hours; neither has interfered with the other; the expanding mind has been a powerful factor in the development of the business, and mind and body have improved under the varied routine of business and pleasure. Pecuniary success and approaching age find such a man ready to leave the counting room with tastes to cater for, and powers to enjoy. Culture, the development of mind and taste, the acquiring of the best that has been said and known, ends only with life itself, and with continued culture come new pleasures; the power of appreciating; the knowledge of discrimination; the eye and other senses have increased powers; beauties are developed on every side which are hidden from, or unappreciated by the uncultured. Such a pursuit fits for excellence in all things, and its influence is felt by all who come within its circle. Whilst such a man is gathering knowledge and acquiring culture, he is also aiding in the refinement and culture of those who surround him.

There is nothing in this pursuit to confine exclusively to the study of books, the every day walks of life, sports, social gatherings, are not without their lessons commingled with pleasures; from the drop of water to the firmament, all that surround us teem with wonders worthy of, and necessary to the inquiries of one who would rank among the cultured.

I have very dimly portrayed or rather hinted at the advantages of culture and the pleasures which attend its pursuit. My object is to awaken the attention of those whose lives are being wasted in pursuits which tend to weaken instead of strengthen the mental powers, and through them sap even life itself.

Unnatural excitement seems to be the end and aim of too many boys, girls, men and women of the present day. I shall not attempt to specify all the different means which are used to produce this result. It is the same enemy to mental improvement, culture and happiness, whether it be in the shape of tobacco, rum, opium, chloral, dime novels or other vices; no man's judgment is strictly sound when under the influence of either. He who finds himself *compelled* to resort to such stimulants has wandered away far from the path which leads to culture and real happiness; to return is difficult.

The pernicious stuff which is circulated broadcast throughout the country, called "cheap literature," exciting stories and chronicles of vice and crime, does immense mischief and is the cause of ruin to many. Go where you will, from parlor to kitchen, from palace to hovel, from the mill to the fields, stowed away in some corner will be found the votary of the sensational novel with nerves excited and eyes distended drinking in the poison which gradually but surely undermines not only the taste, but the capability, for reading any book of profit. The penalty for this course is plain, nervous diseases, the sacrifice of all taste for the ordinary pleasures of life, the acquiring of an entirely false conception of human nature, and errors of judgment which too frequently end in the wreck of life. Man is endowed by the Eternal with rich gifts; shall they brighten his road through life by their use, or shall they lie hidden and unused ? In their use we find the duties of life lightened and its pleasures enhanced; in their neglect or misuse we exist only, and share with the brute creation the privileges of sensual life.

